# Research hotspots and trends of thoracic analgesia from 1994 to 2023: a bibliometric analysis

**DOI:** 10.3389/fsurg.2025.1526426

**Published:** 2025-09-03

**Authors:** Yuxiang Meng, Chenyang Shi, Hengrui Zhang, Zijie Ling, Sumin Yuan, Yang Niu, Li Zhang, Zhibiao Xu, Yuyun Liu, Su Liu, Linlin Zhao

**Affiliations:** Department of Anesthesiology, Affiliated Hospital of Xuzhou Medical University, Xuzhou, China

**Keywords:** anesthesiology, bibliometrics, CiteSpace, thoracic analgesia, nerve block

## Abstract

**Purpose:**

This study employs bibliometric analysis to comprehensively evaluate research trends in thoracic analgesia, identify evolving thematic priorities, and highlight emerging frontiers. The objective is to provide actionable insights to guide future investigations in this field.

**Methods:**

The publications related to thoracic analgesia from the establishment of the Web of Science Core Collection (WOSCC) to 2023 were comprehensively searched. Utilizing CiteSpace (version 6.2.R4) software, this study conducted an in-depth analysis of the included publications, including disciplines, publication years, countries, institutions, authors, journals, cited references and keywords.

**Results:**

A total of 3,895 articles related to thoracic analgesia were included in this study. Anesthesiology, surgery, respiratory system and cardiovascular system are the most active subjects in the study of thoracic analgesia. Since the first article was published in 1994, the number of articles related to thoracic analgesia has been on the rise. It is worth noting that the relevant literature mainly comes from developed countries, especially North America and Europe, of which the United States is far ahead. The analysis of institutions, authors and journals further reveals the important contributions made by the United States in the study of thoracic analgesia. In addition, the analysis of references on the number of citations showed that erector spinae plane block (ESPB), serratus anterior plane block (SAPB) and thoracic paravertebral block (TPVB) were the research hotspots in recent years. The analysis of key words also shows that Video-Assisted Thoracoscopic Surgery (VATS) and nerve block are the development trend in the field of thoracic surgery.

**Conclusion:**

This study highlights the research hotspots and trends in thoracic analgesia, nerve block-related techniques (e.g., ESPB, SAPB) emerged as dominant research hotspots. Future research hotspots will be more centered on the relationship between thoracic analgesia and nerve block.

## 1 Introduction

Thoracic surgery is highly invasive and painful, and more than 70% of patients may experience pain after thoracic surgery (1). Postoperative pain not only brings pain to patients, but also increases stress response, changes endocrine and immune response. Adequate analgesia is essential for patients at high risk of respiratory and cardiac complications (2). Since the publication of the first article on thoracic analgesia in 1994 (3, 4), a large number of articles on analgesic methods for thoracic surgery have been published worldwide each year. These include regional block techniques (such as thoracic epidural block, TPVB, intercostal nerve block, ESPB, etc.), systemic analgesia (such as opioids, non-steroidal anti-inflammatory drugs [NSAIDs], COX-2 inhibitors, etc.), and multimodal analgesia protocols (such as combinations of regional block and systemic medications).

This study used bibliometrics to comprehensively review and analyze the relevant publications. Bibliometrics is a subject that uses mathematical and statistical methods to quantitatively analyze academic publications, so as to provide insights into academic works. In this study, CiteSpace software (5) (version 6.2.R4) was used to analyze the literature related to thoracic analgesia, examining the discipline, year of publication, country, institution, authors, citations, journals, and keywords, with a view to analyzing the current status of development in the field of thoracic analgesia, revealing its research hotspots and trends, and identifying the hot topics of research and the keywords for bursts. To date, there are no bibliometric studies specific to thoracic analgesia.

## 2 Methods

### 2.1 Data sources and search strategy

We utilized the Web of Science Core Collection (WOSCC) (1994-present). The search formula was: ((ALL = (post-thoracotomy analgesia)) OR ALL = (post-thoracic surgery analgesia)) OR ALL = (thoracic analgesia). We excluded articles published in 2024 and included only original articles and review papers.

### 2.2 Analysis

Knowledge graph visualization analysis was carried out utilizing CiteSpace 6.2.R4 software after automatic de-duplication, and in this study, the time setting was adjusted to exclude data from 2024, and the time slice was set to 1 year. Node Types were selected as Discipline, Country, Institution, Author, Journal, Cited References, and Keywords, etc., respectively, to carry out the knowledge graph visualization analysis of the included literature.

## 3 Results

### 3.1 General information

Based on the screening criteria, 3,908 publications were identified for this study, with 3,405 dissertations and 503 review papers. Finally, a total of 3,895 publications between 1994 and 2023 were included in Citespace for analysis. These literature covered 70 disciplines with anesthesiology, surgery, respiratory system, cardiovascular system and clinical neurology being the key areas of interest (Table 1).

**Table 1:** Top 10 disciplines according to literature classification (year indicates the number of years at the time of the first publication).

Count	Centrality	Year	WoS Categories
1,884	0.08	1994	ANESTHESIOLOGY
757	0.15	1994	SURGERY
579	0.04	1994	RESPIRATORY SYSTEM
550	0.02	1994	CARDIAC & CARDIOVASCULAR SYSTEMS
270	0.22	1995	CLINICAL NEUROLOGY
249	0.11	1994	MEDICINE, GENERAL & INTERNAL
249	0.01	1994	BIOCHEMISTRY & MOLECULAR BIOLOGY
245	0.16	1994	VETERINARY SCIENCES
194	0.05	1994	CRITICAL CARE MEDICINE
116	0.02	1995	PEDIATRICS

### 3.2 Year of publication

**Figure 1:**
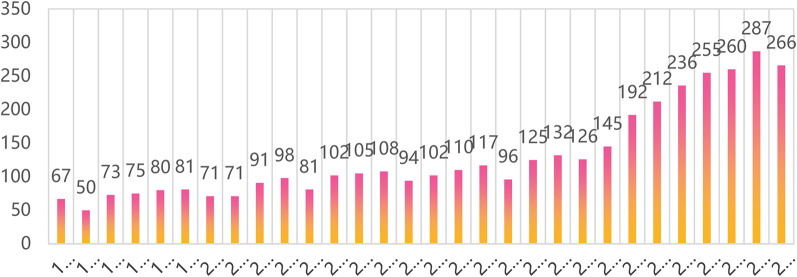
Annual number of publications in the literature related to thoracic analgesia.

This study reveals the stages of development and trends by analyzing the distribution of relevant publications in the field of thoracic analgesia over time. The first article in the field of thoracic analgesia was published in 1994. Figure 1 depicts the number of publications related to thoracic analgesia from 1994 to 2023. These 30 years can be divided into three distinct phases: an initial slow growth phase (1994–2015), followed by a sharp rise (2015–2020), and then stabilization over the past four years (2020–2023). In addition, this can be seen in the rapid increase in global anesthesiology research capacity.

### 3.3 Countries

**Figure 2:**
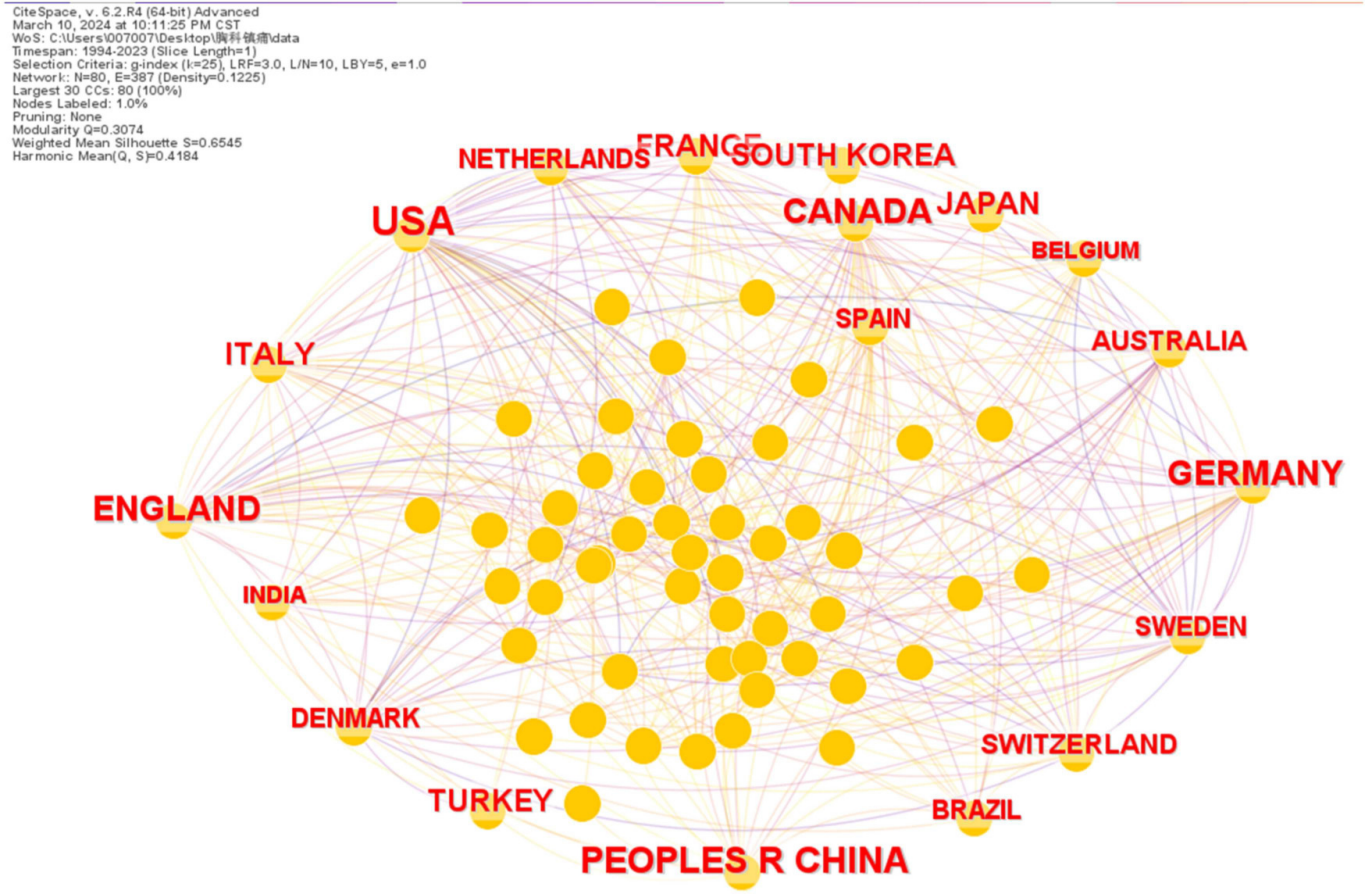
The number of publications by country and collaborative relationships.

Over the 30-year period from 1994 to 2023, the United States (*n* = 1,051) emerged as the leading publishing country, followed by China (*n* = 453), Germany (*n* = 284), the United Kingdom (*n* = 280), and Canada (*n* = 270) (Figure 2). The partnership between each country was quite complex, with the top three countries ranking in terms of centrality of collaboration being: the United States, the United Kingdom, and Canada, and far more than the other countries (Table 2). It can be seen that the United States is leading the research in the field of thoracic analgesia and makes great contributions. On the other hand, China and Germany, which are in the second and third place in terms of the number of publications respectively, have less cooperation with other countries in comparison. Centrality measures the importance of a node (e.g., country, institution, keyword) within the network, often reflecting its role as a bridge or hub connecting different parts of the network. That is, it plays a key role in the information transfer process.

**Table 2:** The top 10 countries ranked by the number of published papers and their centrality.

Rank	Country	Count	Centrality
1	USA	1,051	0.39
2	PEOPLES R CHINA	453	0.02
3	GERMANY	284	0.06
4	ENGLAND	280	0.21
5	CANADA	270	0.20
6	ITALY	176	0.06
7	JAPAN	161	0.00
8	TURKEY	155	0.00
9	FRANCE	146	0.03
10	SOUTH KOREA	128	0.00

USA, United States of America; PEOPLES R CHINA, People's Republic of China.

### 3.4 Institutions

**Figure 3:**
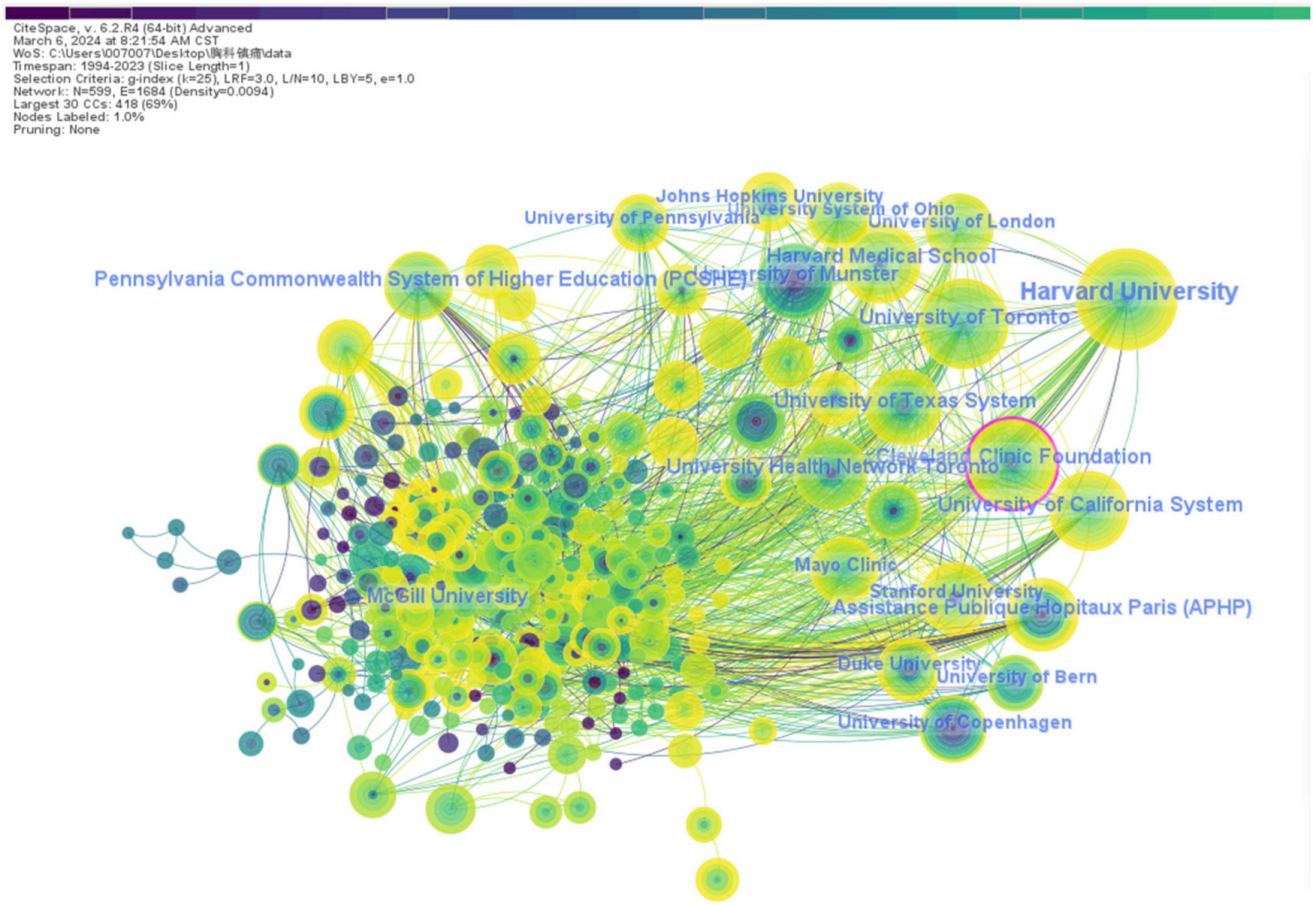
Visualization of the cooperation between the issuing institutions.

Publication-related research institutions were analyzed by CiteSpace software with g-index (*k* = 25), LRF = 3.0, L/N = 10, LBY = 5, *e* = 1.0, where there were a total of 599 research institutions and 1,684 collaborative connections, yielding a visualization of the collaborative relationships between these institutions (Figure 3). The top five institutions in the field in terms of the number of publication were Harvard University (*n* = 94), the University of Toronto (*n* = 76), the University of California system (*n* = 68), Cleveland Medical Center (*n* = 57), and Harvard Medical School (*n* = 52). In addition, the top three institutions with the highest centrality scores were Cleveland Medical Center (0.12), University of California System (0.07), and University of Texas System (0.07). The United States is the absolute leader in both the number of publications and centrality scores.

### 3.5 Authors

**Figure 4:**
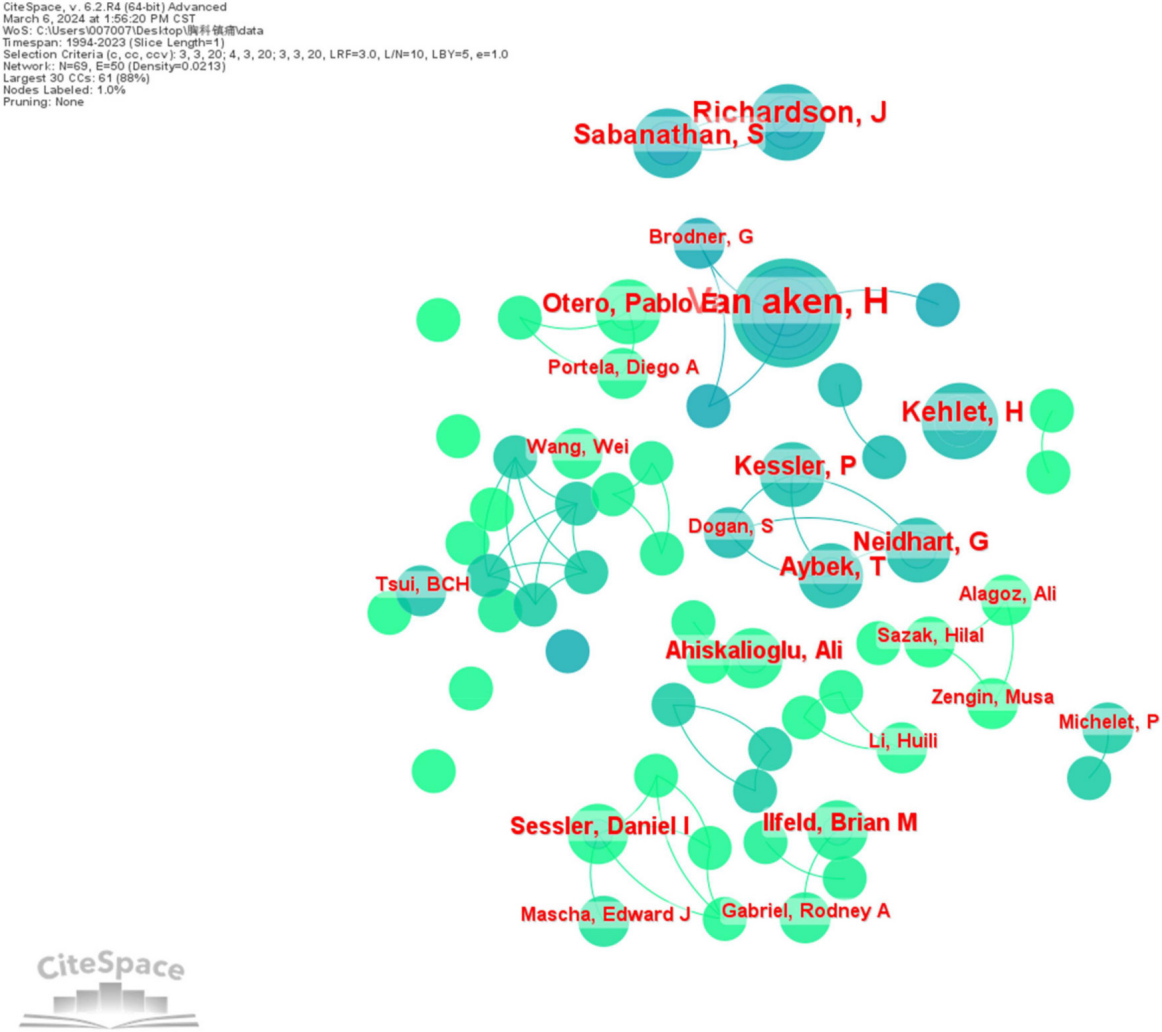
Visualization of collaborative relationships between authors of publications.

Literature published by authors of studies related to thoracic analgesia was analyzed utilizing CiteSpace software. g-index (*k* = 25), LRF = 3.0, L/N = 10, LBY = 5, *e* = 1.0, generating a visual map representing author collaborations (Figure 4). Due to the generally low number of authors' publications, only authors with publications greater than or equal to 3 were selected for mapping in this study, resulting in *N* = 70 and *E* = 50.The results of the study indicate that authors collaborate mainly within their teams or research organizations. Among the authors, the top five contributors with the highest number of publications were VAN AKEN H (*n* = 19), RICHARDSON J (*n* = 10), KEHLET H (*n* = 9), SABANATHAN S (*n* = 8) and AYBEK T (*n* = 7) (Table 3). It is worth noting that although only authors with publications greater than or equal to 3 were selected, in this case all individuals had a centrality score of 0.00, which implies that author collaborations were mainly limited to their respective teams or research institutions.

**Table 3:** Top 10 authors in terms of number of publications (year indicates the number of years at the time of the first publication).

Count	Year	Authors
19	1997	VAN AKEN H
10	1995	RICHARDSON J
9	2000	KEHLET H
8	1995	SABANATHAN S
7	2002	AYBEK T
7	2002	NEIDHART G
7	2002	KESSLER P
7	2018	CHIN KI JINN
7	2020	OTERO PABLO E
6	2019	AHISKALIOGLU ALI

**Figure 5:**
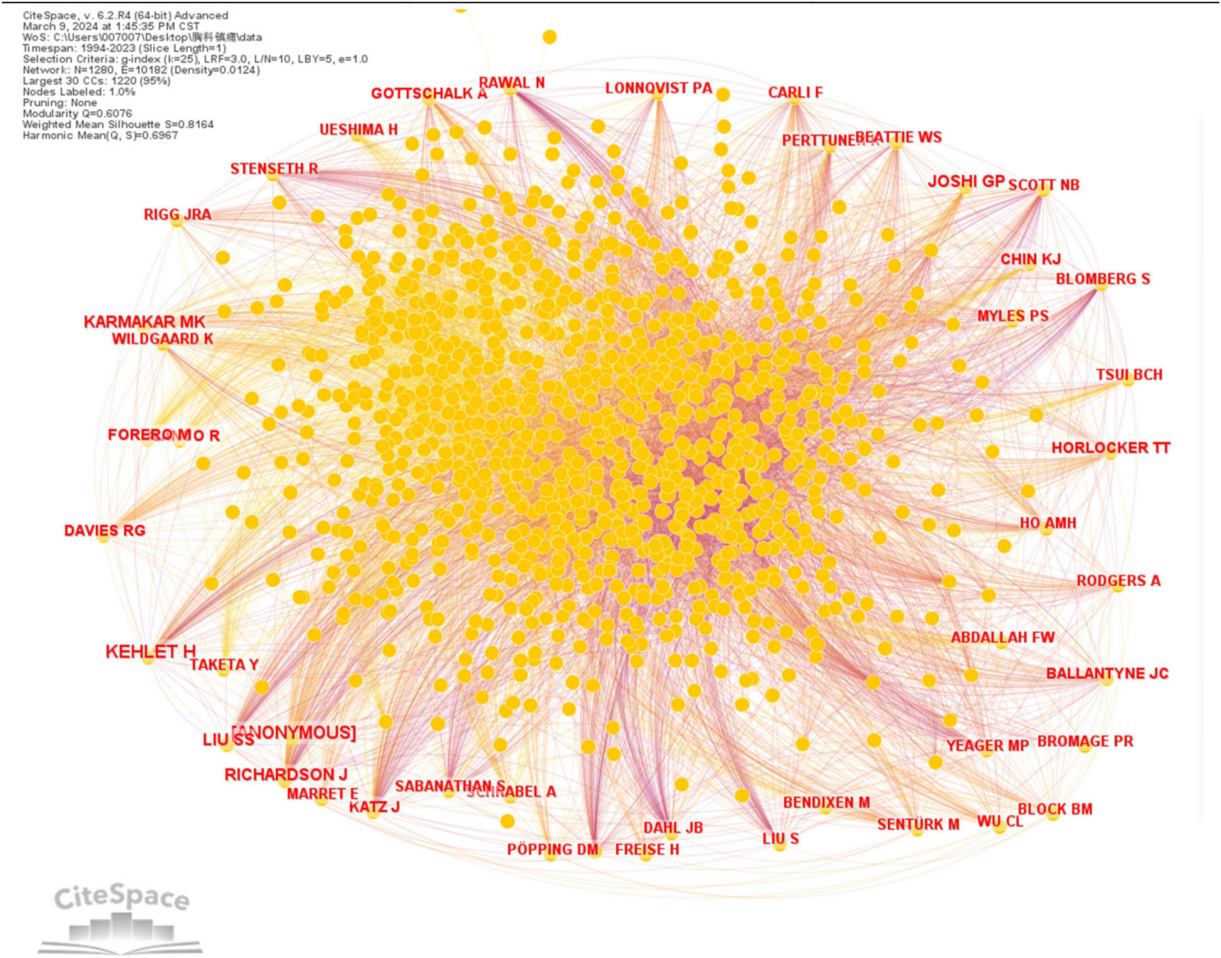
Author co-citation visualization map.

The authors' citations were visualized using CiteSpace software (Figure 5). Among the five most cited authors, KEHLET H, RICHARDSON J, JKARMAKAR MK, LU SS, JOSHI GP (Table 4). Table 5 also provides the ranking of authors according to their centrality in co-citations.

**Table 4:** Top 10 most cited authors.

Rank	Count	Cited Authors
1	487	KEHLET H
2	318	RICHARDSON J
3	290	JKARMAKAR MK
4	265	LU SS
5	247	JOSHI GP
6	236	BLANCO R
7	220	FORERO M
8	219	DAVIES RG
9	217	KATZ J
10	198	BALLANTYNE JC

**Table 5:** Top 10 cited authors by centrality.

Rank	Author	Centrality
1	RICHARDSON J	0.10
2	KATZ J	0.07
3	KEHLET H	0.06
4	BALLANTYNE JC	0.06
5	HORLOCKER TT	0.06
6	SENTURKM	0.06
7	KARMAKAR MK	0.05
8	RAWALN	0.05
9	CARLIF	0.05
10	MOINICHE S	0.05

### 3.6 Cited journals

CiteSpace software was used to analyze the cited journals of the literature related to thoracic analgesia. The top 5 most frequently cited journals were *Anesthesia And Analgesia, Anesthesiology*, *British Journal of Anaesthesia*, *Anaesthesia*, *Acta Anaesthesiologica Scandinavica* (Table 6). These most prestigious journals in the field of anesthesiology have contributed greatly to the study of thoracic analgesia. (Journal citation Indicator and Journal Citation Reports partitioning data are from Web of Science).

**Table 6:** Top 10 co-cited journals by number of citations.

Rank	Co-cited journals	Citations	Country	Journal Citation Indicator	Category Quartile
1	Anesthesia And Analgesia	2,770	USA	1.96	Q1
2	Anesthesiology	2,631	USA	2.87	Q1
3	British Journal of Anaesthesia	2,412	UK	2.82	Q1
4	Anaesthesia	1,675	UK	3.23	Q1
5	Acta Anaesthesiologica Scandinavica	1,556	DENMARK	0.78	Q4
6	Regional Anesthesia And Pain Medicine	1,375	USA	1.78	Q1
7	Annals Of Thoracic Surgery	1,188	USA	1.31	Q1
8	Journal Of Cardiothoracic And Vascular Anesthesia	1,144	USA	0.69	Q3
9	European Journal of Cardio-thoracic Surgery	1,013	Netherlands	1.13	Q1
10	Journal Of Clinical Anesthesia	976	USA	1.62	Q1

USA, United States of America; UK, United Kingdom.

### 3.7 Cited references

**Figure 6:**
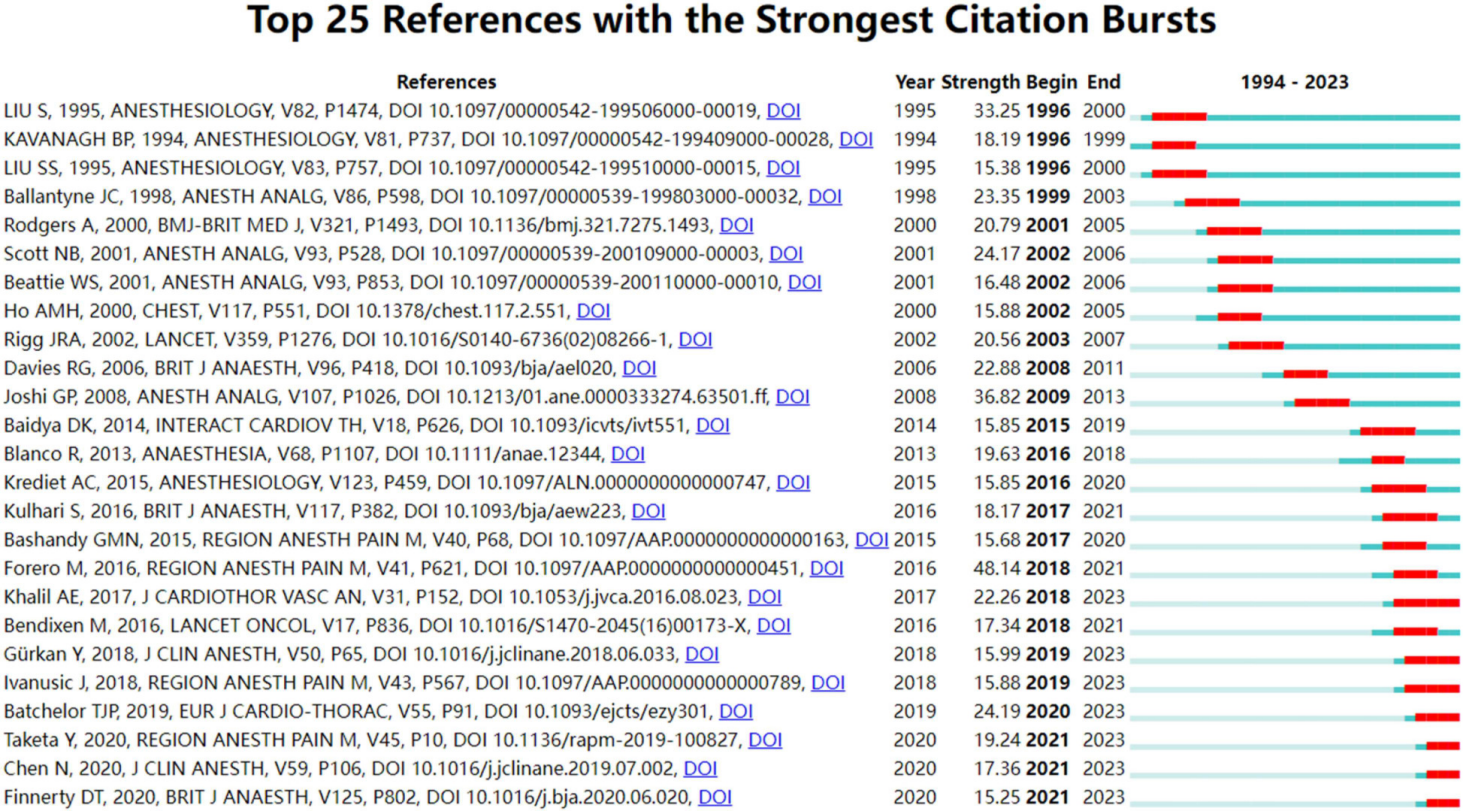
Top 25 references with an explosion of citations.

The cited reference pertains to the source or reference of a particular work or paper utilized during the course of academic research. The frequency of citation within the literature serves as an indicator of the quality and influence of that literature. Of the 3,895 papers included in this study, the top 10 most cited references are shown in Table 7. Burst detection identifies keywords or references that experience a sudden surge in citation frequency over a specific period. The detection is done in CiteSpace utilizing the algorithm proposed by Kleinberg (2002). If a paper suddenly shows a sharp increase in citation frequency, then it means that the paper has hit the key issues in the academic field or it has new insights into solving important problems in the field. Figure 6 shows the top 25 breaking papers in the field of thoracic analgesia.

**Table 7:** Top 10 references cited in 3,895 publications (up to 2023).

Rank	First Author	Year	Count	Journal	DOI
1	Forero et al. (6)	2016	121	Regional Anesthesia And Pain Medicine	10.1097/AAP.0000000000000451
2	Batchelor et al. (7)	2019	88	European Journal Of Cardio-Thoracic Surgery	10.1093/ejcts/ezy301
3	Khalil et al. (8)	2017	76	Journal Of Cardiothoracic And Vascular Anesthesia	10.1053/j.jvca.2016.08.023
4	Ivanusic et al. (9)	2018	67	Regional Anesthesia And Pain Medicine	10.1097/AAP.0000000000000789
5	Adhikary et al. (10)	2018	64	Regional Anesthesia And Pain Medicine	10.1097/AAP.0000000000000798
6	Joshi et al. (11)	2008	62	Anesthesia And Analgesia	10.1213/01.ane.0000333274.63501.ff
7	Taketa et al. (12)	20202	58	Regional Anesthesia And Pain Medicine	10.1136/rapm-2019-100827
8	Gürkan et al. (13)	2018	57	Journal Of Clinical Anesthesia	10.1016/j.jclinane.2018.06.033
9	Kim et al. (14)	2018	56	Anesthesia And Analgesia	10.1213/ANE.0000000000002779
10	Liu et al. (15)	1995	56	Anesthesiology	10.1097/00000542-199506000-00019

### 3.8 Keywords

**Figure 7:**
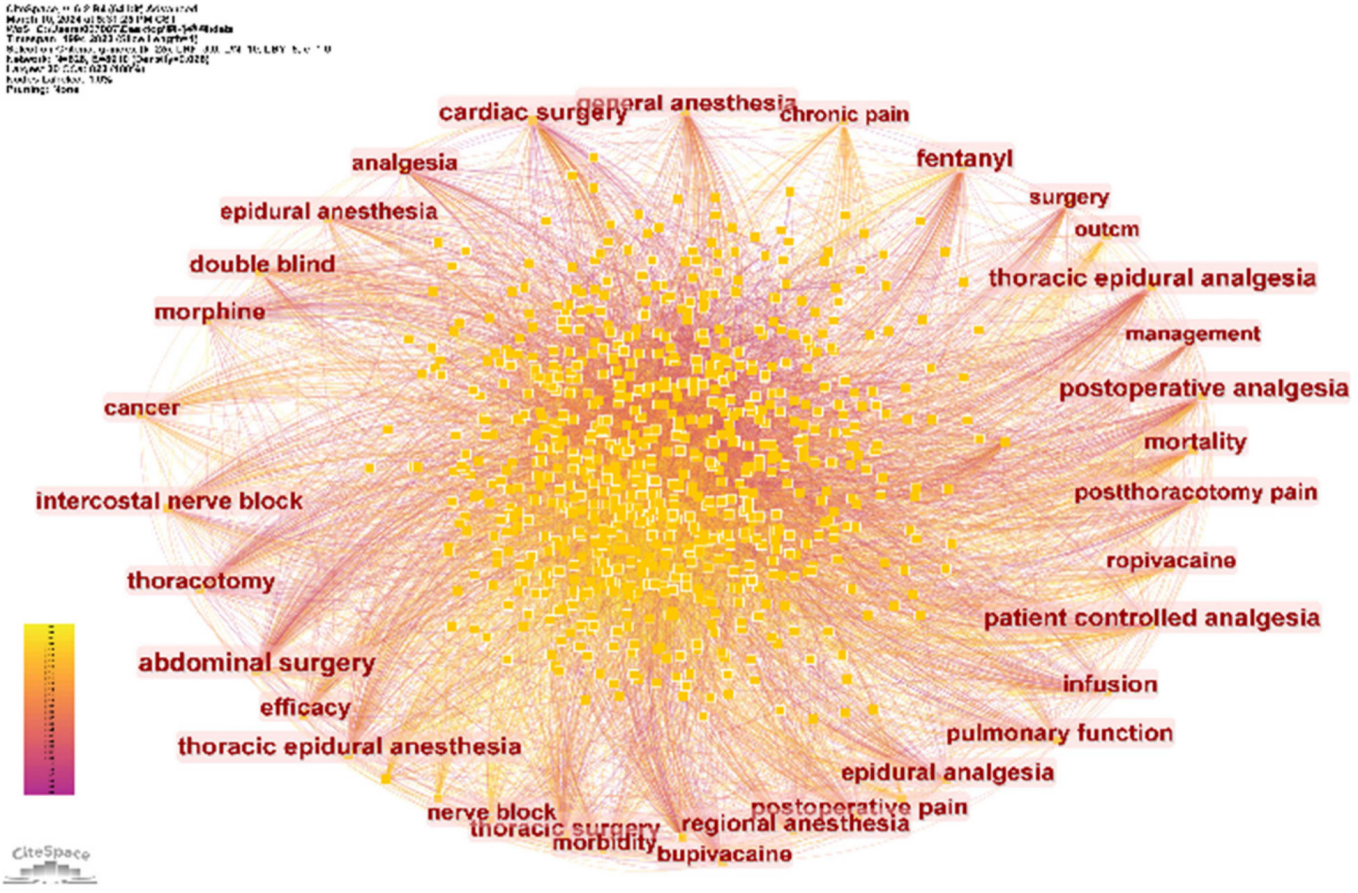
Keyword co-occurrence diagram.

**Figure 8:**
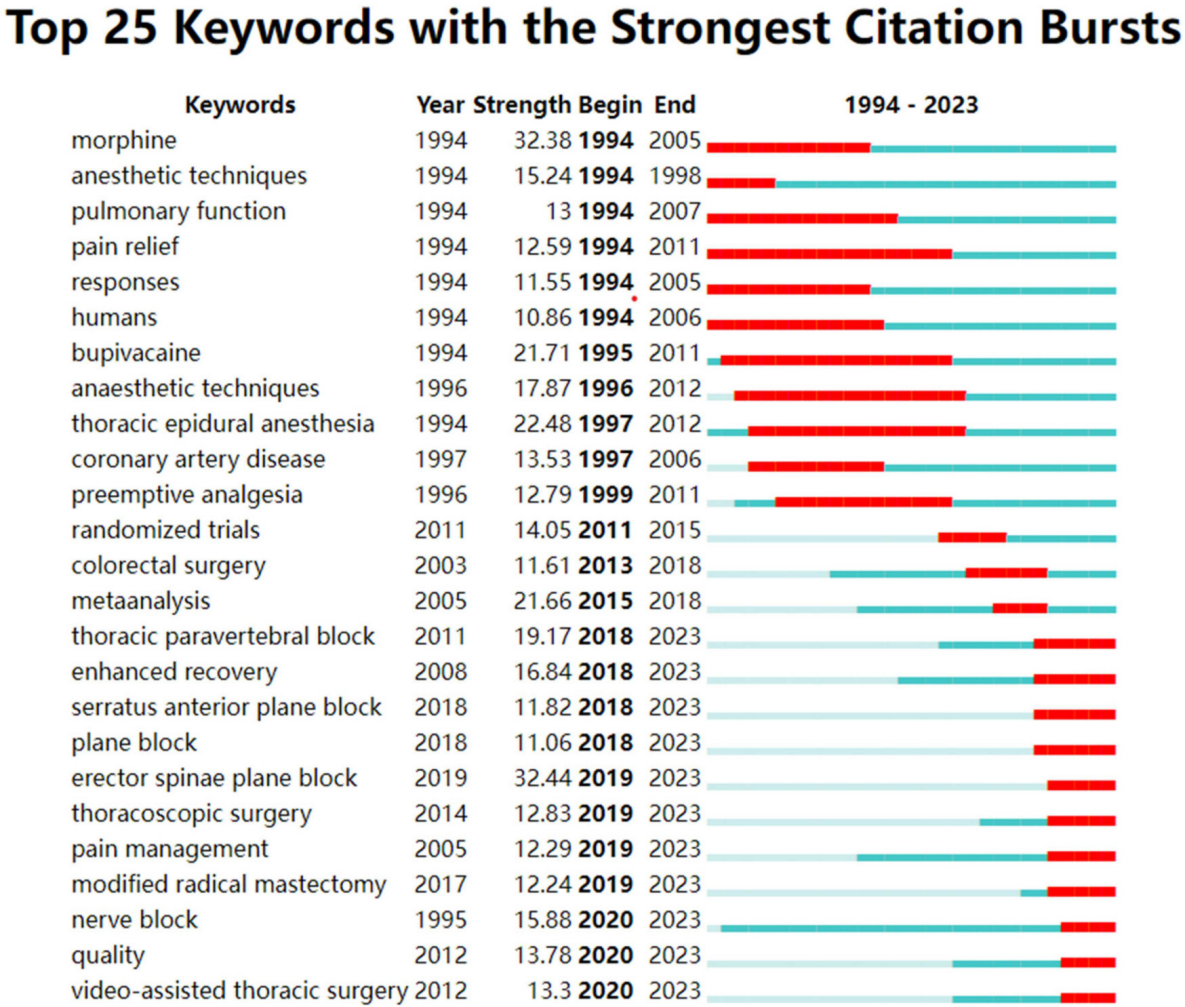
Shows the top 25 keywords with the strongest citation bursts. (Dark blue represents the occurrence and duration of high-frequency keywords, while red represents burst citations and duration of specific keywords).

CiteSpace software was used to analyze keywords in articles related to thoracic analgesia. A total of 828 keywords and 8,910 connections were found. The keyword co-occurrence map is shown in Figure 7. Five of the most commonly used keywords were analgesia, anesthesia, surgery, postoperative pain and thoracic surgery (Table 8). In addition, the top three keywords with the highest centrality scores were abdominal surgery, cardiac surgery, and patient-controlled analgesia (Table 9). By examining the high-frequency keywords over the past 30 years, Figure 8 illustrates the changes and duration of these emerging keywords each year. It is worth noting that some emerging keywords continue until 2023, including thoracic paravertebral block, enhanced recovery, serratus anterior plane block, plane block, erector spinae plane block, thoracoscopic surgery, pain management, modified radical mastectomy, nerve block, quality, video-assisted thoracic surgery.

**Table 8:** Top 10 keywords ranked by frequency of occurrence.

Rank	Keywords	Year	Count
1	Analgesia	1994	903
2	Anesthesia	1994	597
3	Surgery	1994	560
4	Postoperative pain	1994	548
5	Thoracic surgery	1994	439
6	Epidural	1995	432
7	Analgesiamanagement	1994	432
8	Pain	1994	398
9	Postoperative analgesia	1996	366
10	Thoracotomy	1994	363

**Table 9:** Top 10 keywords ranked by centrality.

Rank	Keywords	Year	Centrality
1	Abdominal Surgery	1994	0.09
2	Cardiac Surgery	1994	0.06
3	Patient Controlled Analgesia	1994	0.06
4	Epidural Analgesia	1995	0.05
5	Thoracic Epidural Analgesia	1996	0.05
6	Thoracic Epidural Anesthesia	1994	0.05
7	Mortality	1997	0.05
8	Fentanyl	1994	0.05
9	Anesthesia	1994	0.04
10	Thoracic Surgery	1994	0.04

### 3.9 Keyword clustering

**Figure 9:**
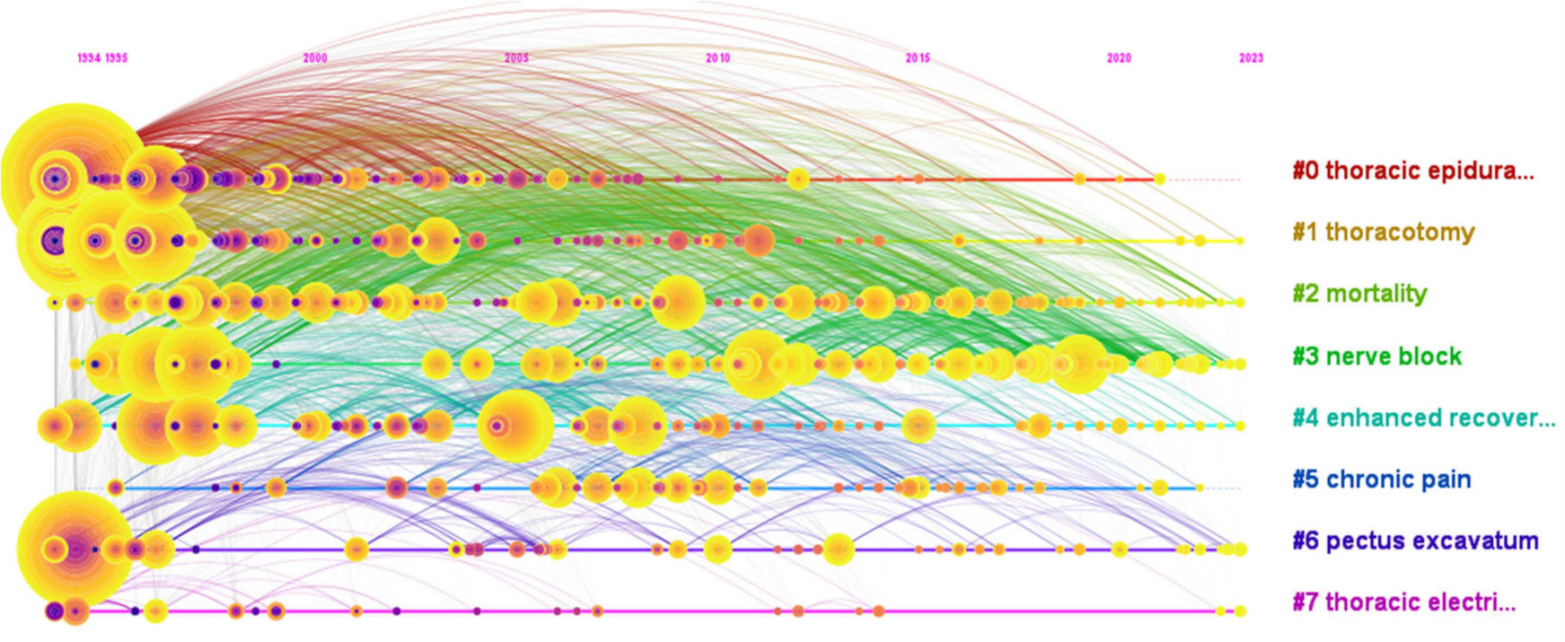
Timeline view of keyword clustering.

Keyword clustering method is a data mining technology based on text analysis, which can classify and cluster texts according to the keywords appearing in the text. This method can help us discover hidden patterns in large amounts of text data. In this study, we use CiteSpace to perform keyword cluster analysis to group keywords with similar themes or concepts, thereby revealing the key research directions and knowledge focus in this field. Figure 9 and Table 10 show the time trend of keyword clustering in the field of thoracic analgesia, including eight keyword clusters: #0 thoracic epidural anesthesia, #1 thoracotomy, #2 mortality, #3 Nerve Block, #4 Enhanced Recovery, #5 Chronic Pain, #6 Pectus Excavatum, and #7 Chest Bioimpedance Monitoring Analysis Technology. Silhouette coefficient is an indicator to evaluate the effectiveness of clustering, and its value ranges from −1 to 1. Values close to 1 indicate better clustering effects, reflecting higher homogeneity of the network, while values close to −1 indicate poor clustering effects. When Silhouette is 0.7, the clustering result is highly reliable. Above 0.5, the clustering results can be considered reasonable. Among the eight keyword clusters, the Silhouette coefficients are all greater than 0.5, which indicates that the clustering effects are good.

**Table 10:** Keyword clustering.

Cluster ID	Cluster label	Size	Silhouette	Mean year
#0	Thoracic Epidural Anesthesia	157	0.659	1999
#1	Thoracotomy	150	0.644	2000
#2	Mortality	147	0.576	2010
#3	Nerve Block	140	0.619	2015
#4	Enhanced Recovery	101	0.639	2007
#5	Chronic Pain	54	0.779	2010
#6	Pectus Excavatum	46	0.805	2010
#7	Thoracic Electrical Bioimpedance	26	0.882	2003

Figure 9 depicts six clusters showing ongoing research activity up to 2023: #1 Thoracotomy, #2 Mortality, #3 Nerve Block, #4 Enhanced Recovery, #6 Pectus Excavatum, and #7 Thoracic Electrical Bioimpedance. While the Silhouette coefficients indicate statistically valid clustering, the clinical relevance of some cluster labels (e.g., #7 Thoracic Electrical Bioimpedance) warrants discussion. This likely reflects a small but distinct set of publications exploring specific monitoring techniques, which may not represent a core clinical theme in thoracic analgesia for most practitioners.

To enhance clinical interpretability, the clusters can be conceptually grouped into broader, more clinically meaningful domains based on their thematic content:

Core Analgesic Techniques & Management:

#0 Thoracic Epidural Anesthesia (Historical gold standard, foundational research)
#3 Nerve Block (Current dominant research focus—ESPB, SAPB,TPVB, etc.)
#4 Enhanced Recovery [Enhanced Recovery After Surgery (ERAS) protocols incorporating multimodal analgesia]

Surgical Context & Outcomes:

#1 Thoracotomy (Traditional surgical approach and its associated pain)
#2 Mortality (Focus on serious outcomes potentially influenced by pain/pain management)

Specific Patient Populations/Problems:

#5 Chronic Pain (Addressing long-term consequences of thoracic surgery)
#6 Pectus Excavatum (Pain management specific to this corrective surgery)

Emerging/Niche Monitoring/Techniques:

#7 Thoracic Electrical Bioimpedance (Representing research on this specific monitoring modality, potentially related to fluid management or respiratory effort, but less central to mainstream analgesic strategy development).

## 4 Discussions

Bibliometrics is a discipline that uses quantitative methods to study the phenomena and regularity of the emergence, structure, transmission and utilization of all kinds of articles. it has been firmly established as a science specialty and is a part of research and evaluation methods, especially in the field of science and application (16).Through the analysis and interpretation of articles data, bibliometrics can help scientists grasp the focus of the field, obtain accurate cutting-edge information, identify research trends and hot spots, and guide further research (17, 18).

Bibliometrics analysis can use CiteSpace software to generate visual maps to reflect the research hotspots and development trends in a certain field. In this study, we searched the WOSCC database for articles related to thoracic analgesia, and applied specific screening criteria, and finally included 3,895 articles. The articles spanned 30 years (from 1994 to 2023) and involved 1,077 authors from 80 countries and 599 institutions. This study makes an in-depth analysis of the included articles data, including disciplines, year of publication, countries, institutions, authors, journals, cited references and key words, etc.

Through the analysis of the discipline, we found that anesthesiology, surgery, respiratory system, cardiovascular system and clinical neurology are the top five areas of thoracic analgesia. Based on the analysis of the year of publication, we find that the development of thoracic analgesia can be divided into three stages: the initial slow development stage (1994–2015), the subsequent sharp rise stage (2015–2020) and the stable stage (2020–2023).

In addition, this study also predicts that the annual number of articles related to thoracic analgesia may exceed 300 in the next three years, because thoracic analgesia will still be an area of interest to scientific researchers in the foreseeable future. From a national point of view, the USA has the most articles, followed by China, Germany, the United Kingdom and Canada. Although China has published a lot of articles and made a great contribution to the field of thoracic science, according to the analysis of its centrality and the cooperative relationship between the authors, there is a serious lack of communication and cooperation between China and other countries. The main exchanges and cooperation exist between developed countries such as North America and Europe.

Regarding hospital institutions, the top five institutions with the highest number of publications are Harvard University (*n* = 94), University of Toronto (*n* = 76), University of California System (*n* = 68), Cleveland Medical Center (*n* = 57), and Harvard Medical School (*n* = 52). Additionally, the top three institutions with the highest centrality scores were Cleveland Medical Center (0.12), University of California System (0.07), and University of Texas System (0.07).VAN AKEN H from the Department of Anesthesiology, Intensive Care Medicine and Pain Management at the University Hospital Munster in Germany have published the most articles, and RICHARDSON J from the Department of Anesthesia at Bradford Royal Hospital has the highest centrality score. KEHLET H is the most cited author from the Department of Cardiovascular and Thoracic Surgery at the University of Copenhagen, with 487 times. An analysis of cited journals related to thoracic analgesia shows that the top five most frequently cited journals are *Anesthesia And Analgesia*, *Anesthesiology*, *British Journal of Anaesthesia*, *Anaesthesia*, and *Acta Anaesthesiologica Scandinavica* (Table 6). These most renowned journals in the field of anesthesiology have made significant contributions to the research of thoracic analgesia.

The 25 papers with the highest burst of citations were analysed, and these high-burst-citation papers showed a sudden sharp increase in citation frequency generally lasting 3–5 years, and these papers invariably hit the key issues in the academic field or their new insights into solving the important problems in the field. Seven of these high sudden citation continues until 2023, namely Khalil et al. (8) on ultrasound-guided anterior serratus muscle plane block with thoracic segmental epidural analgesia for open-heart surgery pain, Gürkan et al. (13) on ultrasound-guided erector spinae muscle plane block to reduce postoperative opioid consumption after breast surgery as randomised controlled study, Ivanusic et al. (9) on the use of Cadaveric injection of dye to simulate erector spinae muscle block and determine dye diffusion, Batchelor et al. (7) on surgical guidelines for accelerated rehabilitation after lung surgery, Taketa et al. (12) on comparison of ultrasound-guided ESPB and TPVB for postoperative analgesia in televised thoracoscopic surgery, Chen et al. (19) on ultrasound-guided intercostal nerve block, single-injection ESPB and multiple-injection paravertebral block for postoperative analgesia in thoracoscopic surgery, and Finnerty et al. (20) on comparison of ESPB with anterior serratus plane block in minimally invasive thoracic surgery.

Pain after thoracic surgery is usually severe and standardized multimodal analgesia strategies are needed to keep patients comfortable. The prominence of 'Enhanced Recovery’ as a distinct and persistent cluster (#4) underscores a major shift in perioperative care philosophy, aligning closely with ERAS principles. To accelerate rehabilitation, multimodal analgesia must be combined with regional analgesia or local anesthesia, while opioids and their side effects should be avoided as far as possible (7). The selection of the most appropriate regional technique is paramount and increasingly guided by the principle of matching the block's anatomical coverage to the primary sources of pain. Research highlights the differential effectiveness of various blocks for specific surgical incisions and drain locations. For example, the Pecto-intercostal Fascial Plane Block (PIFB) has shown particular promise for median sternotomy pain and anterior chest tube sites (21), whereas SAPB may be more suited for lateral thoracotomy incisions or VATS (VATS) ports/drains placed laterally (8), and ESPB/TPVB for posterior/lateral approaches (6). This granular approach to selecting regional techniques based on anticipated pain generators is a key advancement facilitated by the proliferation of ultrasound-guided plane blocks. Landmark guidelines, such as those jointly published by the ERAS Society and the European Society of Thoracic Surgeons (ESTS), have been pivotal in standardizing and promoting these principles for lung surgery. These guidelines explicitly prioritize regional analgesic techniques (like TPVB, and by extension, newer blocks such as ESPB and SAPB) as fundamental components of multimodal, opioid-sparing strategies within ERAS pathways. The strong recommendations within these guidelines (e.g., advocating regional analgesia over systemic opioids alone) have provided a clear framework and impetus for research focusing on optimizing the integration, efficacy, and implementation of nerve blocks specifically within the context of accelerated recovery protocols. Consequently, the sustained bibliometric activity observed in both the “Nerve Block” (#3) and “Enhanced Recovery” (#4) clusters is mutually reinforcing, reflecting this essential synergy in contemporary thoracic analgesia research.

Epidural analgesia is still considered as the “gold standard” technique for first-line analgesia and pain control after thoracic surgery ^(^11),our bibliometric findings [e.g., rise of #3 Nerve Block, high burst citations for SAPB/ESPB studies (8, 12)] reveal a significant and ongoing shift towards TPVB and novel plane blocks (ESPB, SAPB). This evolution in preferred techniques is underpinned by accumulating comparative effectiveness evidence. Meta-analyses, such as the seminal work by Davies et al. (22) (also among our highly cited references, Table 7), provided early and compelling evidence that TPVB could achieve analgesia comparable to thoracic epidural analgesia (TEA) while potentially reducing the incidence of troublesome side effects like hypotension, urinary retention, and nausea/vomiting. TPVB is therefore gradually considered to be the closest method to epidural. Subsequent high-quality RCTs comparing newer blocks (SAPB, ESPB) directly against both TEA [e.g., Khalil et al. (8)] and TPVB [e.g., Taketa et al. (12), as captured by our burst detection analysis (Figure 6), have further expanded the evidence base, demonstrating non-inferiority or even specific advantages (e.g., technical ease, applicability in anticoagulated patients) for these newer approaches, particularly in the context of minimally invasive surgery. Compared with TEA, TPVB (and increasingly, plane blocks) reduces the risk of mild complications… There was no difference in acute pain, 30-day mortality, major complications… or hospital stay (11, 22). Collectively, this body of evidence challenges the absolute dominance of TEA and highlights the growing recognition and research focus on effective, potentially safer regional alternatives, a trend vividly reflected in our bibliometric data.

A recent meta-analysis found that the use of acetaminophen in thoracic surgery reduced morphine consumption by 20%, but did not change the incidence of postoperative morphine-related adverse reactions (23). Thoracoscopic surgery has been widely used because of its advantages such as fewer postoperative complications, less postoperative pain, shorter hospital stay, and better quality of life compared with traditional thoracotomy (24, 25). Because epidural analgesia is associated with potential risks of dural puncture, nerve injury, epidural hematoma, and hypotension, it is not recommended for pain control after VATS. Coupled with recent reductions in postoperative opioid use and associated side effects, regional anesthesia techniques are playing an increasingly important role in multimodal analgesia. As early as 2016, a prospective, randomized, observer-blinded, controlled study published in the Journal Of Cardiothoracic And Vascular Anesthesia pointed out that the SAPB appears to be an effective and safe alternative to TEA after thoracotomy (8). A prospective randomized non-inferiority trial published in *Regional Anesthesia And Pain Medicine* pointed out that the analgesic effect of ESPB 24 h after VATS was not inferior to TPVB (12). A randomized clinical controlled trial published in *British Journal of Anaesthesia* found that ESPB provided better 24-h recovery quality and lower morbidity after minimally invasive thoracic surgery compared with SAPB rate and better analgesic effect (20). Although SAPB and ESPB have been proven to relieve post-thoracic pain, their applicability and specific dosage still need to be proven by large-scale randomized controlled trials.

The bibliometric analysis consistently identifies nerve block techniques, particularly ESPB, SAPB, and TPVB, as dominant research hotspots in recent years (Figure 6, Table 8, Cluster #3). This intense focus is strongly supported by landmark randomized controlled trials (RCTs) demonstrating their efficacy and safety profile compared to traditional methods. For instance, Khalil et al. (8) provided robust evidence that SAPB could serve as a viable alternative to TEA for thoracotomy pain, offering comparable analgesia with a potentially favorable side-effect profile. Similarly, Taketa et al. (12) established the non-inferiority of ESPB to TPVB for analgesia after VATS, a finding that significantly boosted interest in ESPB due to its perceived technical simplicity. Finnerty et al. (20) further refined the comparison, suggesting ESPB might offer advantages over SAPB in terms of recovery quality and analgesia for minimally invasive procedures. These pivotal trials, reflected in our citation burst analysis (Figure 6), have been instrumental in driving the widespread research interest and clinical exploration of these novel regional techniques. With the development of ultrasound visualization technology and technological advancement, it is expected that thoracic nerve blocks will become more and more widely used for postoperative analgesia in thoracic surgery. With the development of ultrasound visualization technology and technological advancement, it is expected that thoracic nerve blocks will become more and more widely used for postoperative analgesia in thoracic surgery.

Based on the analysis of keywords, this study lists the 10 keywords with the most frequent occurrence (Table 8) and the top 10 keywords with center degree (Table 9). This study also collates 25 strong keywords (Figure 8). The high-burst citation of morphine began in 1994 and ended in 2005, while the high-burst citation of bupivacaine began in 1994 and ended in 2011. New key words such as thoracic paravertebral block, enhanced recovery, serratus anterior plane block, plane block, erector spinae plane block, thoracoscopic surgery, pain management, modified radical mastectomy, nerve block, quality, video-assisted thoracic surgery and so on began to appear in 2018, and continues to this day. It can be seen that nerve block is a hot topic and research trend in recent years. In order to show the changes of the theme of thoracic analgesia research in the past 30 years, the key words were analyzed by cluster analysis.

The keyword cluster analysis (Table 10, Figure 9) yielded eight distinct themes. While statistically robust (Silhouette > 0.5), the clinical focus of these clusters varies. To better align with clinical practice perspectives, we propose grouping the clusters into broader domains: 1. core Analgesic Techniques & Management (#0 Thoracic Epidural Anesthesia, #3 Nerve Block, #4 Enhanced Recovery), 2. surgical Context & Outcomes (#1 Thoracotomy, #2 Mortality), 3. specific Patient Populations/Problems (#5 Chronic Pain, #6 Pectus Excavatum), and 4. Emerging/Niche Monitoring/Techniques (#7 Thoracic Electrical Bioimpedance). This grouping reveals that the most intense and sustained research activity, particularly the clusters persisting until 2023 (#3 Nerve Block, #4 Enhanced Recovery), falls squarely within Core Analgesic Techniques & Management. The timeline view (Figure 9) further illustrates the evolution: Thoracic epidural analgesia (#0) dominated the early years (1994–2012), establishing itself as the historical gold standard. The pivotal shift occurred around 2010 with the rapid ascent of #3 Nerve Block, driven by advancements in ultrasound guidance. This cluster encompasses the explosion of research on novel blocks like ESPB, SAPB, and TPVB, which continues unabated. Concurrently, #4 Enhanced Recovery emerged, emphasizing multimodal protocols and accelerated rehabilitation, becoming intricately linked with optimizing regional techniques like nerve blocks. The sustained activity in these two clusters (#3 and #4) underscores their status as the current and future central axis of thoracic analgesia research. Research within the Surgical Context & Outcomes (#1, #2) and Specific Patient Populations/Problems (#5, #6) domains provides essential context and addresses important complications or subgroups, but the driving force and primary focus remain on refining and implementing effective regional analgesic strategies within ERAS pathways. Cluster #7, while identifiable, represents a much narrower technological thread within the broader field.

This is the first study to analyze the articles related to thoracic analgesia in the past 30 years, using CiteSpace software which provides valuable insights for researchers in the field of thoracic analgesia. Besides CiteSpace, VOSviewer, and Bibliometrix, commonly used tools include Gephi, VantagePoint, built-in analysis tools in Web of Science/Scopus, and R packages. CiteSpace was chosen for several key advantages: its unique time-slicing function visualizes the 30-year evolutionary trajectory of thoracic analgesia research, precisely capturing historical milestones and emerging trends through longitudinal dynamic analysis. It integrates multifunctional capabilities—such as co-citation analysis, keyword burst detection, and collaboration network mapping—in a single platform, balancing analytical depth with operational efficiency. Its timeline and cluster visualizations intuitively link conceptual networks to historical contexts, while burst detection highlights pivotal hot topics, critical for fields with rapid technological advancements like thoracic analgesia.

However, our research has some inherent limitations.

1. Database Coverage: Our exclusive reliance on WOSCC constitutes the primary limitation. While WOSCC is prominent for bibliometric studies, it does not provide exhaustive global coverage. Crucially, significant research indexed in other major databases (e.g., Scopus, PubMed, Embase) or published in regionally influential journals, particularly non-WOSCC-indexed or non-English publications, may have been excluded. This affects dataset comprehensiveness and potentially introduces geographic/journal representation bias.
2. Search Strategy Specificity: Despite using comprehensive search terms for thoracic analgesia, some relevant articles utilizing alternative terminology or indexing may have been missed.
3. Inherent Bibliometric Scope: This analysis, like all bibliometric studies, is constrained by its source data and cannot encompass all existing literature or unpublished research. We acknowledge valuable contributions that may not be represented due to database coverage or terminology limitations.

## 5 Conclusions

Since the first thoracic analgesia study in 1994, research has grown annually, spanning stages and focusing on fields like anesthesiology and surgery, with the U.S., Harvard University, and authors such as VAN AKEN H and KEHLET H leading contributions across publications, citations, and collaboration metrics. Key journals like *Anesthesia And Analgesia* have promoted its development, while recent research, driven by compelling evidence from landmark randomized trials (8, 12, 20) and clinical guidelines [e.g., ERAS®/ESTS (7)], highlights nerve block—notably erector spinal and anterior serratus muscle blocks—and their integration within enhanced recovery protocols for postoperative recovery. Despite this progress, challenges remain in understanding pain mechanisms and tailoring individualized analgesia plans due to subjective pain metrics and individual variability in sensitivity.
